# Down-regulation of miR-223 reverses epithelial-mesenchymal transition in gemcitabine-resistant pancreatic cancer cells

**DOI:** 10.18632/oncotarget.2714

**Published:** 2015-02-02

**Authors:** Jia Ma, Binbin Fang, Fanpeng Zeng, Cong Ma, Haijie Pang, Long Cheng, Ying Shi, Hui Wang, Bin Yin, Jun Xia, Zhiwei Wang

**Affiliations:** ^1^ The Cyrus Tang Hematology Center and Collaborative Innovation Center of Hematology, Jiangsu Institute of Hematology, The First Affiliated Hospital, Soochow University, Suzhou 215123, China; ^2^ Department of Biochemistry and Molecular Biology, Bengbu Medical College, Anhui 233030, China; ^3^ Research Center of Clinical Laboratory Science, Bengbu Medical College, Anhui 233030, China; ^4^ Department of Clinical Laboratory, Yijishan Hospital, Wannan Medical College, Wuhu 241000, Anhui, China

**Keywords:** Gemcitabine, miR-223, EMT, invasion, pancreatic cancer

## Abstract

Recent studies have demonstrated that acquisition of epithelial-to-mesenchymal transition (EMT) is associated with drug resistance in pancreatic cancer cells; however, the underlying mechanisms are not fully elucidated. Emerging evidence suggests that microRNAs play a crucial role in controlling EMT. The aims of this study were to explore the potential role of miR-223 in governing EMT in gemcitabine-resistant (GR) pancreatic cancer cells. To achieve this goal, real-time reverse transcription-PCR and western blot analysis were used to validate whether GR cells acquired EMT in AsPC-1 and PANC-1 cells. Invasion, migration, and detachment assays were performed to further identify the EMT characteristics in GR cells. The miR-223 inhibitor was used to determine its role in GR-induced EMT. We found that GR cells acquired EMT features, which obtained elongated fibroblastoid morphology, decreased expression of epithelial marker E-cadherin, and up-regulation of mesenchymal markers. Furthermore, we observed that GR cells are associated with high expression of miR-223. Notably, inhibition of miR-223 led to the reversal of EMT phenotype. More importantly, miR-223 governs GR-induced EMT in part due to down-regulation of its target Fbw7 and subsequent upregulation of Notch-1 in pancreatic cancer. Our study implied that down-regulation of miR-223 could be a novel therapy for pancreatic cancer.

## INTRODUCTION

Pancreatic cancer (PC) is one of lethal malignant tumors with high morbidity and mortality. Approximately 46,420 new cases and 39,590 deaths were expected to occur from PC in 2014 [1]. It has been well known that one of the reasons for the aggressiveness of PC was due to its intrinsic and extrinsic drug resistance to chemotherapy [2]. The standard chemotherapy was gemcitabine alone or in combination with other chemo-therapeutic agents for advanced PC patients [3, 4]. Although chemotherapy and systemic treatments have improved effective therapies for PC patients, the overall 5-year survival rate of PC is less than 6% [1]. Thus, it is vital to explore the molecular mechanism of drug resistance to gemcitabine, which could help us to find a promising strategy for the treatment of PC.

Emerging evidence has demonstrated that epithelial-to-mesenchymal transition (EMT) plays an essential role in the progression of PC [5]. It is known that during EMT process, epithelial cells acquire mesenchymal phenotype, resulting in enhanced invasion and metastasis [6]. Concomitantly, epithelial cells loss the expression of epithelial markers such as E-cadherin, whereas cells obtain higher expression of mesenchymal markers including Vimentin, Snail, Slug, zinc-finger E-box binding homeobox 1 (ZEB1) and ZEB2 [7]. There is growing evidence that EMT is associated with drug resistance [8, 9]. For example, the transcription factor Twist1, one of EMT inducers, has been found to be involved in tumor metastasis and chemoresistance in ovarian cancer cells [10]. Furthermore, it has been shown that erlotinib resistance associated with EMT is due to dysregulation of Steroid receptor or coactivator/focal adhesion kinase (Src/FAK) pathway in non-small cell lung cancers [11]. Moreover, gemcitabine resistance is associated with EMT and induction of platelet-derived growth factor D (PDGF-D) and HIF-1alpha (HIF-1α) in PC cells [12–14].

Multiple studies have indicated that microRNAs (miRNAs) was critically involved in regulation of drug resistance-mediated EMT [15]. It has been found that up-regulation of miR-200 and let-7 led to the reversal of EMT in gemcitabine-resistant PC cells [16]. Similarly, another study identified that miR-200 expression regulates EMT in bladder cancer cells and reverses resistance to epidermal growth receptor therapy [17]. Moreover, the expression levels of miR-200c and its target, mitogen-inducible gene 6, are highly correlated with EMT and resistance to erlotinib [18]. Similarly, miR-200c counteracts trastuzumab resistance and metastasis through targeting zinc finger gene 217 (ZNF217) and ZEB1 in breast cancer [19]. Furthermore, miR-200b and miR-15b regulate cisplatin-induced EMT by targeting B lymphoma Mo-MLV insertion region 1 homolog (BMI1) in human tongue cancer cells [20]. Additionally, re-expression of miR-375 was found to sensitize tamoxifen resistance and reverse EMT in tamoxifen resistant breast cancer cells [21]. Notably, miR-365 was found to induce gemcitabine resistance in PC cells via targeting apoptosis-promoting protein BAX and Src homology 2 domain containing 1 (SHC1) [22]. Chang et al. reported that overexpression of let-7d effectively reversed the EMT and increased the chemosensitivity in oral cancer cells, whereas down-regulation of let-7d increased chemo-resistant abilities of oral cancer cells [23]. These findings suggest the important role of miRNAs in regulation of chemotherapy-induced EMT. However, whether miR-223 is involved in regulating chemotherapy-induced EMT in human cancer remains unclear.

In the present study, we explored the role of miR-223 in regulating gemcitabine-induced EMT. We established stable gemcitabine-resistant PC cell lines AsPC-1 gemcitabine resistance (GR) and PANC-1 GR. We found that AsPC-1 GR and PANC-1 GR cells displayed mesenchymal features and acquired increased motility and invasiveness. Moreover, we revealed that miR-223 was highly expressed in both GR cells. More importantly, we identified that downregulation of miR-223 led to the reversal of EMT phenotype and inhibition of migration and invasion in GR cells. Therefore, miR-223 could be a novel therapeutic target to reverse chemotherapy resistance in PC.

## RESULTS

### Establishment of gemcitabine-resistant cell lines

**Figure 1:**
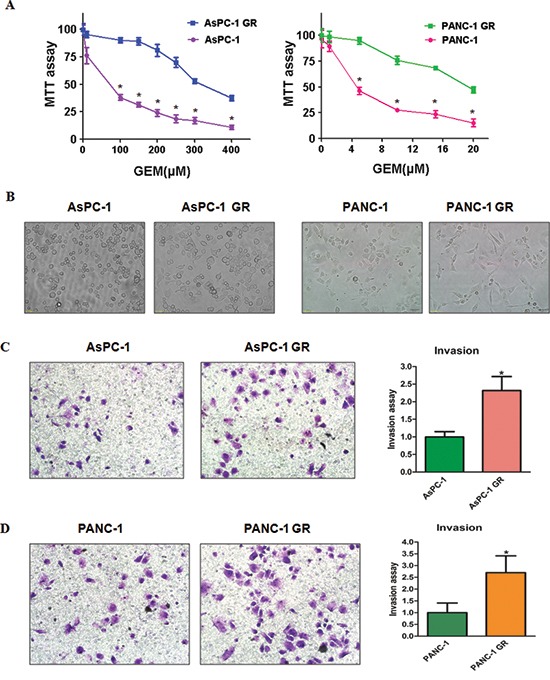
Gemcitabine-resistant (GR) cells exhibited EMT phenotype **(A)** MTT assay was conducted in parental and GR pancreatic cancer cells. *P < 0.05 Parental cells vs GR cells. **(B)** Cell morphology was observed by microscopy in parental and GR cells. Parental AsPC-1 and PANC-1 cells displayed an epithelioid appearance, whereas their GR cells showed elongated, irregular fibroblastoid morphology. **(C-D)** Left panel: Invasion assay was performed to measure the invasive capacity in AsPC-1 GR **(C)** and PANC-1 GR **(D)** cells. Right panel: Quantitative results are illustrated for left panel. *P < 0.05 vs control.

To determine the mechanism of drug resistance in PC, we created the gemcitabine-resistant (GR) PC cell lines. The AsPC-1 and PANC-1 cells were treated with increasing concentrations of gemcitabine for more than 6 months. We observed that 100μM gemcitabine treatment caused about 65% growth inhibition in AsPC-1 cells (Figure 1A). Similarly, 5μM gemcitabine treatment led to about 50% growth inhibition in PANC-1 cells (Figure 1A). These findings suggest that these two PC cell lines exhibited different sensitivity to gemcitabine. However, 100μM gemcitabine and 5μM gemcitabine did not cause the cell growth inhibition in AsPC-1 GR and PANC-1 GR, respectively. The resistant cells were continuously maintained in culture medium containing gemcitabine for the following study.

### GR cells show EMT characteristics

Previous studies have demonstrated that BxPC-3 GR cells acquired EMT characteristics [13, 24]. In line with this notion, we observed that AsPC-1 GR and PANC-1 GR cells exhibited EMT phenotype. Specifically, we observed the markedly morphologic changes associated with EMT feature in GR cells (Figure 1B). As shown in Figure 1B, AsPC-1 and PANC-1 cells displayed a rounded shape, whereas GR cells exhibited elongated, fibroblastoid morphology. Consistently, we found that GR cells have significantly increased numbers of invaded cells through a Matrigel-coated membrane compared with parental cells (Figure 1C, 1D), suggesting that GR cells obtained enhanced invasion.

### GR cells acquire EMT feature

**Figure 2:**
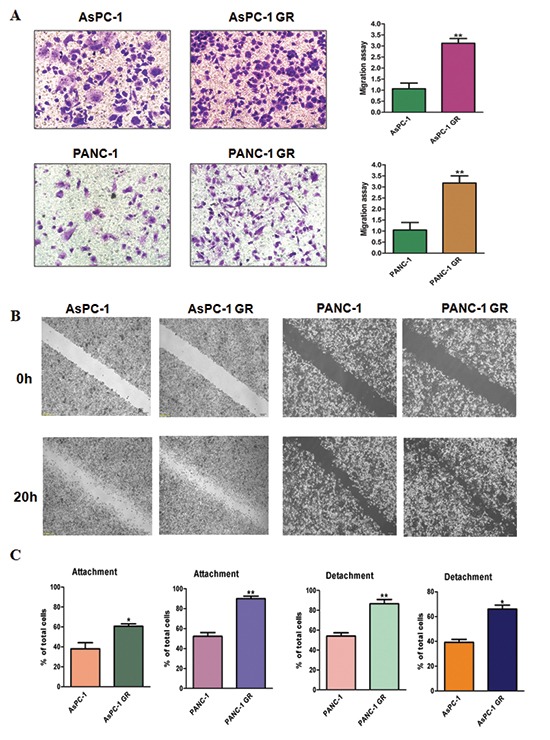
Gemcitabine-resistant (GR) cells have enhanced motility activity **(A)** Left panel: migration assay was performed in parental and GR cells. Right panel: Quantitative results are illustrated for left panel. *P < 0.05 vs control. **(B)** Wound assays were performed in parental and GR cells. **(C)** Cell attachment and attachment assays were assessed in parental and GR cells. *P < 0.05 vs control.

It is known that after EMT, cells have increased migration and metastasis. To explore whether GR cells have aggressive characteristics, we measured the migration in parental cells and GR cells. Our migration results showed that GR cells have increased migration compared with their parental cells (Figure 2A). To further validate whether GR cells have enhanced motility, we detected cell migration using wound-healing assay. We found that GR cells have higher numbers of cells migrating across the wound (Figure 2B). In line with these findings, we identified that GR cells have increased attached and detached activities (Figure 2C).

### GR cells have EMT molecular marker changes

**Figure 3:**
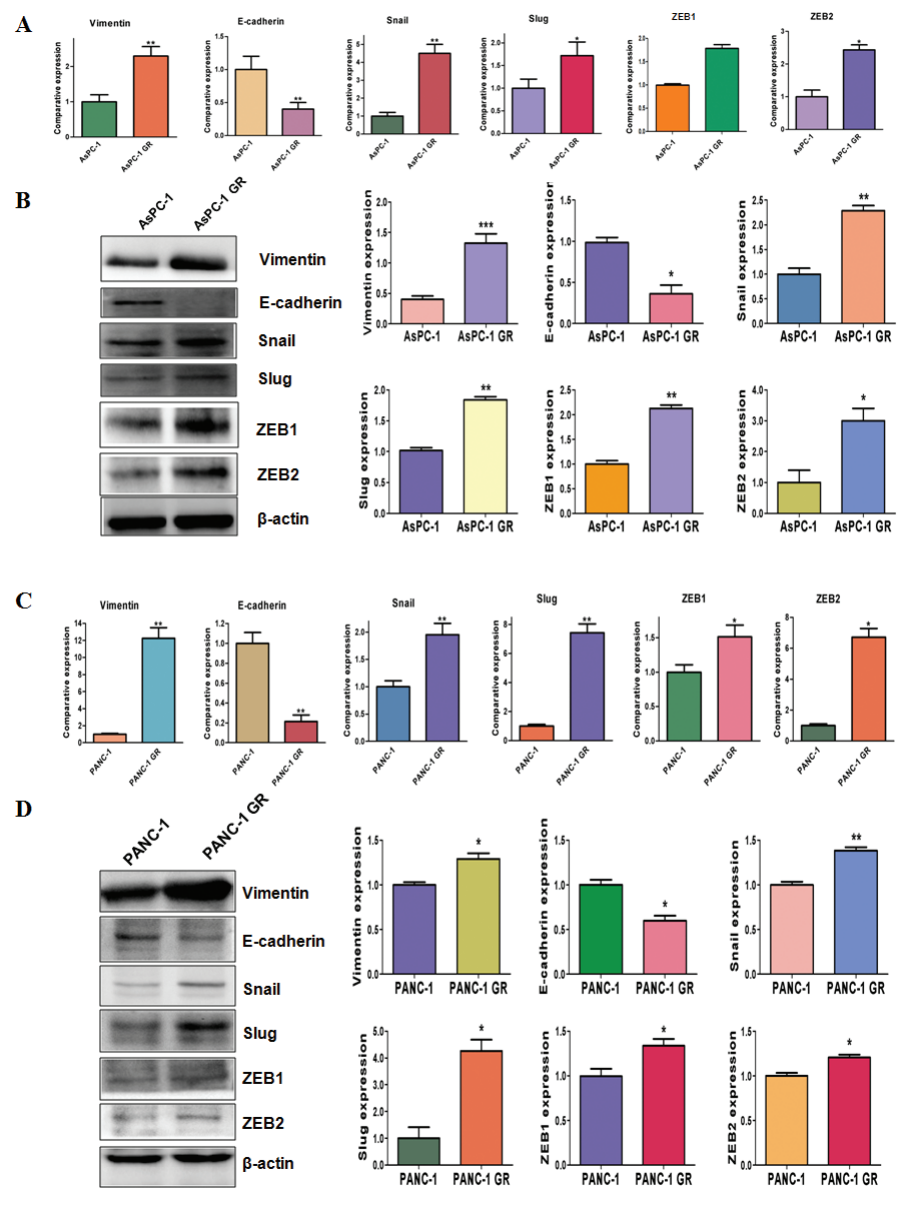
Gemcitabine-resistant (GR) cells have EMT marker changes **(A)** Real-time RT-PCR assay was conducted to detect the expression of EMT markers in AsPC-1 and AsPC-1 GR cells. *P < 0.05 GR vs control. **(B)** Left panel: Western blotting analysis was used to detect the expression of Vimentin, E-cadherin, Snail, Slug, ZEB1 and ZEB2 in AsPC-1 and AsPC-1 GR cells. Right panel: Quantitative results are illustrated for left panel. *P < 0.05 vs control. **(C)** Real-time RT-PCR assay was used to measure the mRNA levels of EMT markers in PANC-1 and PANC-1 GR cells. *P < 0.05 GR vs control. **(D)** Western blotting analysis was performed to measure the expression of EMT markers in PANC-1 and PANC-1 GR cells. Right panel: Quantitative results are illustrated for left panel. *P < 0.05 vs control.

To identify whether GR cells have EMT molecular marker changes, we measured the mRNA levels of EMT markers using RT-PCR in paired parental cells and GR cells. We found that epithelial molecule E-cadherin mRNA was down-regulated, while the mRNA levels of mesenchymal markers including Vimentin, Snail, Slug, ZEB1 and ZEB2 were up-regulated in GR cells (Figure 3A and 3C). To validate whether the protein levels of EMT markers have changes, we determined the expression of these EMT molecules by Western blotting analysis. Consistent with RT-PCR results, Western blotting analysis showed that GR cells have decreased E-cadherin expression, but higher expression of mesenchymal markers (Figure 3B and 3D). These results further suggest that GR cells acquired a mesenchymal phenotype.

### High expression of miR-223 is found in GR cells

**Figure 4:**
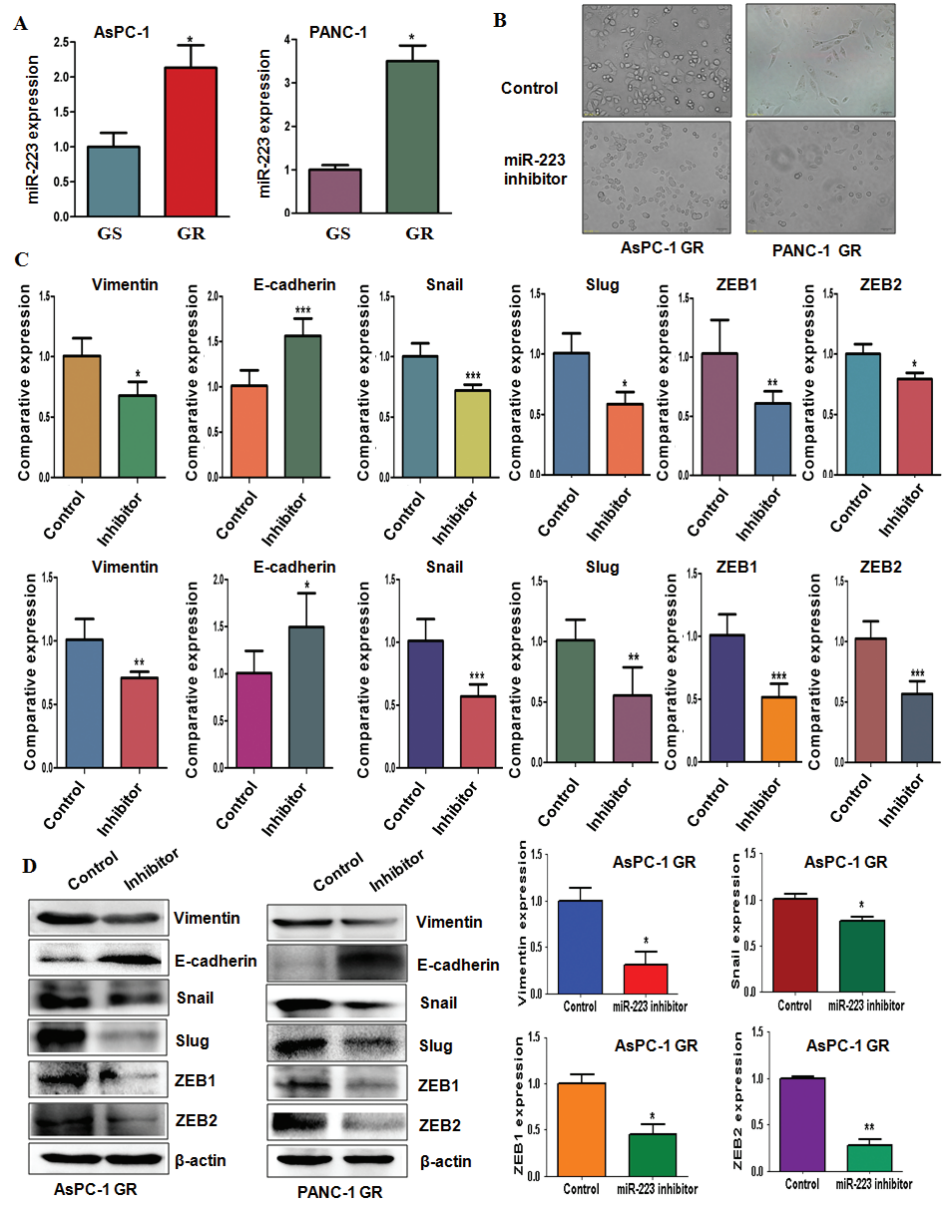
Gemcitabine-resistant (GR) cells have high expression of miR-223 **(A)** TaqMan miRNA assay was conducted to detect the expression of miR-223 in parental and GR cells. *P < 0.05 GR vs control. **(B)** Cell morphology was taken by microscopy in GR cells transfected with miR-223 inhibitor. **(C)** Real-time RT-PCR analysis was performed to detect the mRNA levels of EMT markers in AsPC-1 GR cells (Top panel) and PANC-1 GR cells (Bottom panel) after miR-223 inhibitor treatment. **(D)** Left panel: Western blotting analysis was performed to detect the expression of EMT markers in AsPC-1 GR and PANC-1 GR cells after miR-223 inhibitor treatment. Right panel: Quantitative results are illustrated for left panel. *P < 0.05 vs control.

Our previous study has demonstrated that miR-223 plays a pivotal role in regulation of cell growth, apoptosis, migration and invasion in PC cells [25]. To define whether miR-223 is involved in GR-induced EMT in PC cells, we detected the expression of miR-223 in GR cells and their parental cells. We observed that miR-223 was highly expressed in GR cells (Figure 4A), suggesting that GR-mediated EMT could be partly due to over-expression of miR-223.

### Inhibition of miR-223 reverses EMT in GR cells

To further determine the role of miR-223 in EMT-type GR cells, we inhibited the expression of miR-223 by its specific inhibitor. We found that miR-223 inhibitor treatment caused round cell-like morphology in AsPC-1 GR and PANC-1 GR cells (Figure 4B). Moreover, we examined the expression of EMT molecules in GR cells transfected with miR-223 inhibitor by real-time RT-PCR and Western blotting analysis. We found that the expression of E-cadherin was significantly increased in GR cells after inhibition of miR-223, whereas the expression of mesenchymal markers including Vimentin, Snail, Slug, ZEB1 and ZEB2 was markedly deceased in GR cells with miR-223 inhibitor treatment (Figure 4C and 4D, Supplementary Figure 1). These findings identified that down-regulation of miR-223 could reverse EMT to MET in GR cells.

### Inhibition of miR-223 reduces cell motility and invasion in GR cells

**Figure 5:**
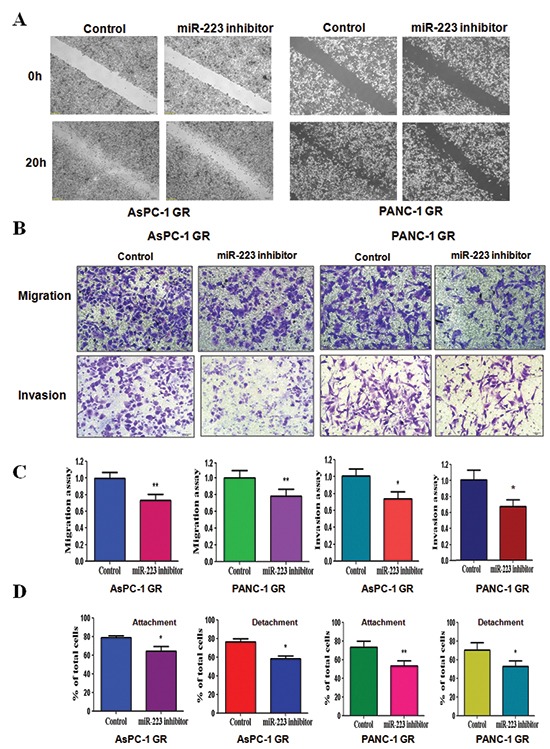
Down-regulation of miR-223 inhibits motility and invasion in gemcitabine-resistant (GR) cells **(A)** Wound healing assays were used to detect the motility in GR cells transfected with miR-223 inhibitor. **(B)** Migration assay (Top panel) and invasion assay (Bottom panel) were conducted in GR cells transfected with miR-223 inhibitor. **(C)** Quantitative results are illustrated for panel B. *P < 0.05, **P < 0.01 vs control. **(D)** Cell attachment and detachment assays were measured in GR cells transfected with miR-223 inhibitor. *P < 0.05, **P < 0.01 vs control.

To further confirm the function of miR-223 in GR cells, we measured the cell motility and invasion capacities in GR cells treated with miR-223 inhibitor. The wound healing assay demonstrated that miR-223 inhibitor reduced cell motility in GR cells (Figure 5A). Consistent with this result, inhibition of miR-223 significantly retarded the migration and invasion in GR cells (Figure 5B and 5C). Furthermore, miR-223 inhibitor treatment reduced the capacity of attachment and detachment in GR cells (Figure 5D). These findings revealed that miR-223 is critically involved in cell migration and invasion characteristics in GR cells. Altogether, our results revealed that miR-223 plays an important role in regulation of EMT in GR cells.

### Downregulation of Fbw7 and overexpression of Notch-1 were found in GR cells

**Figure 6:**
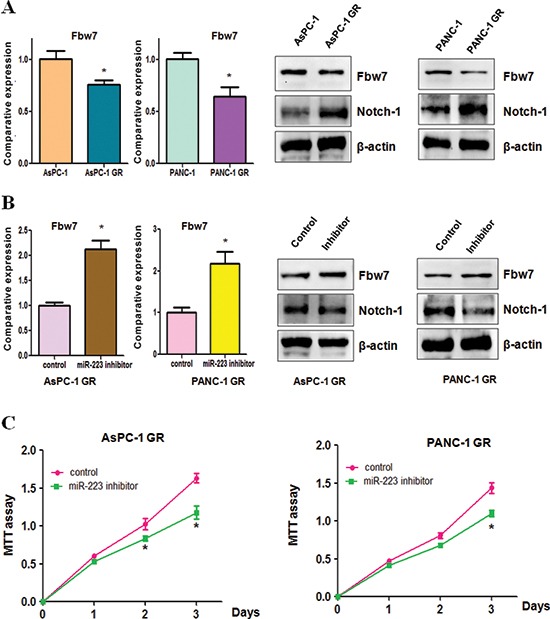
Gemcitabine-resistant (GR) cells have decreased Fbw7 and increased Notch-1 expression **(A)** Real-time RT-PCR analysis was performed to detect the mRNA levels of Fbw7 in parental cells and GR cells (Left panel). Western blotting analysis was used to detect the expression of Fbw7 and Notch-1 in GR cells (Right panel). *P < 0.05 GR vs control. **(B)** Real-time RT-PCR was conducted to detect Fbw7 mRNA level in GR cells after miR-223 inhibitor treatment (Left panel). The expression of Fbw7 and Notch-1 was determined by Western blotting analysis in GR cells treated with miR-223 inhibitor (Right panel). *P < 0.05 vs control. **(C)** MTT assay was performed in GR cells treated with miR-223 inhibitor. *P < 0.05 vs control.

Recently, emerging evidence has demonstrated that F-box and WD repeat domain-containing 7 (Fbw7) is one of miR-223 targets [26, 27]. To further define whether Fbw7 plays a key role in GR-mediated EMT, we measured the expression of Fbw7 at mRNA and protein levels in GR cells and their parental cells using real-time RT-PCR and Western blotting, respectively. We observed that the expression of Fbw7 at mRNA and protein was markedly decreased in GR cells compared with parental cells (Figure 6A). Consistently, the expression of Fbw7 substrate Notch-1 was significantly increased (Figure 6A). Moreover, we found that miR-223 inhibitor treatment led to upregulation of Fbw7 in AsPC-1 GR and PANC-1 GR cells (Figure 6B). Furthermore, Notch-1 expression was downregulated in GR cells treated with miR-223 inhibitor (Figure 6B). These findings indicated that the acquisition of EMT could be in part due to down-regulation of Fbw7 and subsequent overexpression of Notch-1 in GR cells.

### Down-regulation of miR-223 enhances GR cells to gemcitabine sensitivity

To determine whether down-regulation of miR-223 enhances GR cells to gemcitabine sensitivity, we performed MTT assay in GR cells treated with miR-223 inhibitor. We observed that inhibition of miR-223 significantly attenuated cell growth inhibition induced by 100μM gemcitabine in AsPC-1 GR and 5μM gemcitabine in PANC-1 GR, respectively (Figure 6C). These results suggested that GR cells with miR-223 inhibitor treatment were significantly more sensitive to gemcitabine-induced cell growth inhibition.

## DISCUSSION

A wealth of evidence has emerged that miR-223 plays an oncogenic role in the development and progression of human cancers. In support of the function of miR-223, it has been observed that overexpression of miR-223 was found and correlated with tumor metastasis and poor prognosis in colorectal cancer patients [28, 29]. Moreover, upregulated miR-223 was also responsible for the poorer prognosis of gastric cancer [30]. Strikingly, p27 modulated miR-223 expression, leading to proper regulation of cell cycle exit in breast cancer cells [31]. Notably, Huang et al. found that miR-223 promoted cell growth and invasion by targeting tumor suppressor paired box 6 (PAX6) in glioblastoma cels [32]. In line with these reports, our previous study has also shown that inhibition of miR-223 suppressed cell growth and induced apoptosis in PC cells [25], indicating that miR-223 acted as an onco-miRNA in PC. Recently, a case-control study that included 409 patients with PC and 25 with chronic pancreatitis plus 312 healthy participants has identified that 38 miRNAs including miR-223 were significantly dysregulated in PC patients compared with controls [33]. Consistently, miR-223 was found to be aberrantly upregulated in PC [34], suggesting that miR-223 could be a novel biomarker for detection of PC. In the current study, we, for the first time, reported that miR-223 plays a crucial role in regulating gemcitabine-induced EMT in PC cells, suggesting that miR-223 could be a potential therapeutic target for PC.

A line of evidence has suggested that miR-223 was associated with resistance to chemotherapeutic treatments. For example, miR-223 was reported to be critically involved in mutant p53-mediated drug resistance [35]. Mutant p53 proteins downregulated miR-223 expression through binding to miR-223 promoter and reduced its transcriptional activity and subsequently upregulated stathmin-1 oncoprotein that is known to confer resistance to chemotherapeutic drugs [35]. In line with this, Yang et al. found that miR-223 modulated multidrug resistance through downregulation of ATP-binding cassette sub-family B member 1 (ABCB1) in hepatocellular carcinoma cells [36]. Overexpression of miR-223 increased sensitivity to anticancer drugs, whereas downregulation of miR-223 had the opposite effect [36]. Similarly, upregulation of miR-223 decreased the sensitivity of gastric cancer cells to trastuzumab, while inhibition of miR-223 restored this trastuzumab sensitivity through regulation of Fbw7 in gastric cancer [37]. In support of these findings, our current study revealed that miR-223 was highly expressed in gemcitabine resistant PC cells, demonstrating that miR-223 is involved in drug resistance in PC.

Recent studies have highlighted the important role of miRNAs in chemotherapy-induced EMT. For instance, miR-489 regulated chemoresistance via EMT pathway in breast cancer [38]. Moreover, it has been reported that miR-134/487b/665 cluster governed transforming growth factor-β (TGF-β)-mediated EMT and drug resistance to gefitinib by targeting membrance associated guanylate kinase inverted 2 (MAGI2) in lung cancer cells [39]. Furthermore, Shien et al. unraveled that acquired resistance to EGFR inhibitors was associated with EMT features in part due to downregulation of miR-200c in cancer cells [40]. Interestingly, miR-216a/217-induced EMT promoted drug resistance via targeting phosphatase and tensin homolog (PTEN) and mothers against decapentaplegic homolog 7 (SMAD7) in hepatocellular carcinoma [41]. In support of the role of miRNAs in regulating chemotherapy-induced EMT, our study identified that miR-223 could control the GR-mediated EMT in PC cells. Recently, it has been revealed that Fbw7 regulated EMT in non-small cell lung cancers and enhanced cisplatin cytotoxicity [42]. In line with this, we found the lower expression of Fbw7 in GR cells with EMT phenotype, demonstrating that Fbw7 could be involved in GR-induced EMT in PC cells. It has been reported that Notch-1 induced EMT and was associated with drug resistance in a variety of human cancers including PC [43, 44]. Consistently, Notch-1 was highly expressed in GR cells, indicating that Notch-1 could play an important role in GR-induced EMT in PC cells.

In summary, our findings unraveled that GR cells underwent EMT in part due to overexpression of miR-223. Moreover, inhibition of miR-223 reversed GR-induced EMT to MET. More importantly, miR-223 governs GR-induced EMT partly due to down-regulation of its target Fbw7 and subsequent upregulation of Notch-1 in PC. This study indicated that inactivation of miR-223 could be a novel strategy for restoring sensitivity to gemcitabine. Our previous study has shown that natural agent genistein downregulated miR-223 expression, leading to inhibition of cell growth, migration and invasion in PC cells [25]. Due to non-toxic nature, inactivation of miR-223 by genistein could be a safer approach for the treatment of PC.

## MATERIALS AND METHODS

### Cell culture, reagents and antibodies

The human pancreatic AsPC-1 and PANC-1 cells were cultured in RPMI 1600 (Invitrogen, Carlsbad, CA, USA) and DMEM (Gibco, Gaithersburg, MD, USA), respectively, supplemented with 10% fetal bovine serum (FBS) in 5% CO_2_ at 37^o^C. AsPC-1 and PANC-1 cells were exposed to escalating concentrations of gemcitabine for 6 months to create gemcitabine-resistant cell lines AsPC-1 GR and PANC-1 GR. MTT [3-(4,5-dimethythiazol- 2-yl)-2,5-diphenyl tetrazolium bromide] was purchased from Sigma (St. Louis, Mo, USA). Antibodies against Vimentin, E-cadherin, Snail, Slug, ZEB1, ZEB2, Fbw7, β-actin and the secondary antibodies were obtained from Santa Cruz Biotechnology (Santa Cruz, CA, USA). Transwell inserts and Matrigel were purchased from BD Biosciences.

### MTT assay

The cells were seeded into 96-well plates (5×10^3^ cells/well) for overnight incubation. Then, cells were treated with different final concentrations for 72h. MTT assay was performed as described earlier [45].

### Transwell migration and invasion assay

The transwell migration and invasion assays were conducted using a 24-well plate with 8-mm pore size chamber inserts (Corning, New York, NY, USA) as described previously [46]. Briefly, the invasion assay was performed using Transwell inserts precoated with Matrigel (BD Biosciences), while the migration assay used non-coated inserts. Cells were seeded into the upper chamber of the insert, which were suspended in serum-free culture medium. Then, medium with 10% fetal bovine serum was added into the lower chamber. After incubation for 24h, the cells in upper chamber were carefully scraped with cotton buds. The migrated and invaded cells on the bottom surface of chambers were fixed with 4% paraformaldehyde for 20 min, and stained with Giemsa solution. The stained cells were imaged and counted in 5 fields with random choice.

### Wound healing assay

The cells were seeded in 6-well plates until the cells reached to more than 95% confluence. Then, the wounded scrape was made using a pipette tip and the consequently generated floating cells were removed using PBS. The wound healing was photographed at 0 hour and 20 hours as described before [47].

### Cell attachment and detachment assay

Cell attachment and detachment assays were performed as previously described [13]. Briefly, the cells were seeded in 24-well plate (5×10^4^ cells/well). For attachment assay, after one hour incubation, non-attached cells were washed twice with PBS, and the attached cells were counted after trypsinization. The attachment data were quantified as a percentage of cell numbers of the attached cells to total cells. For detachment assay, the cells were detached with 0.05% trypsin for 3 minutes and counted after the seeded cells were incubated 24 hours. The remaining attached cells were further trypsinized with 0.25% trypsin and counted. Cells detachment data were expressed as a percentage of the detached cells to total cells.

### Real-time RT-PCR (RT-PCR)

The total RNA was obtained from cells using Trizol Reagent (Invitrogen, CA, USA) following the manufacturer's protocol. The miR-223 expression was analyzed using the TaqMan miRNA assays (Applied Biosystems, CA, USA) according to the manufacturer's instructions. The expression level was normalized using U6 snRNA levels. The expression of EMT associated markers, such as E-cadherin, Snail, Slug, Vimentin, ZEB1 and ZEB2, was determined using SYBR green RT-PCR assay (Takara, Dalian, China) and normalized to GAPDH as previously published [48]. The primers used in PCR reaction are described before [12, 46]

### Western blotting analysis

The cells were washed twice with PBS and lysed with RIPA buffer supplemented with protease inhibitors. The protein concentrations were detected using the BCA protein assay. The proteins were separated using SDS–PAGE electrophoresis and transferred to PVDF membranes. The membranes were then blocked with 5% nonfat milk and immunoblotted with antibodies as early published [49].

### miRNA-223 inhibitor tranfection

The cells were seeded in six-well plates and transfected with miR-223 inhibitor or the nonspecific control (GenePharma, shanghai, china) using DharmaFect Transfection Reagent (Dharmacon, CO) following the manufacture's protocol [47]. MiR-223 inhibitor: 5′-UGG GGU AUU UGA CAA ACU GAC A-3′. The cells were subjected to further analysis as presented under the results section.

### Statistical analysis

Statistical comparisons were analyzed by Student's t-test using GraphPad Prism 4.0 (Graph pad Software, La Jolla, CA) because we only compared with two different groups. The results were presented as means ± SEM. *P* < 0.05 was considered statistically significant.

## SUPPLEMENTARY FIGURE


