# Supplementary material for: Down-regulation of miR-223 reverses epithelial-mesenchymal transition in gemcitabine-resistant pancreatic cancer cells
Source: Oncotarget. 2015 Feb 2;6(3):1740–9. doi: 10.18632/oncotarget.2714 (PMC4359328; doi:10.18632/oncotarget.2714)
Supplement: Supplementary file 1 [file oncotarget-06-1740-s001.pdf]

SUPPLEMENTARY FIGURE

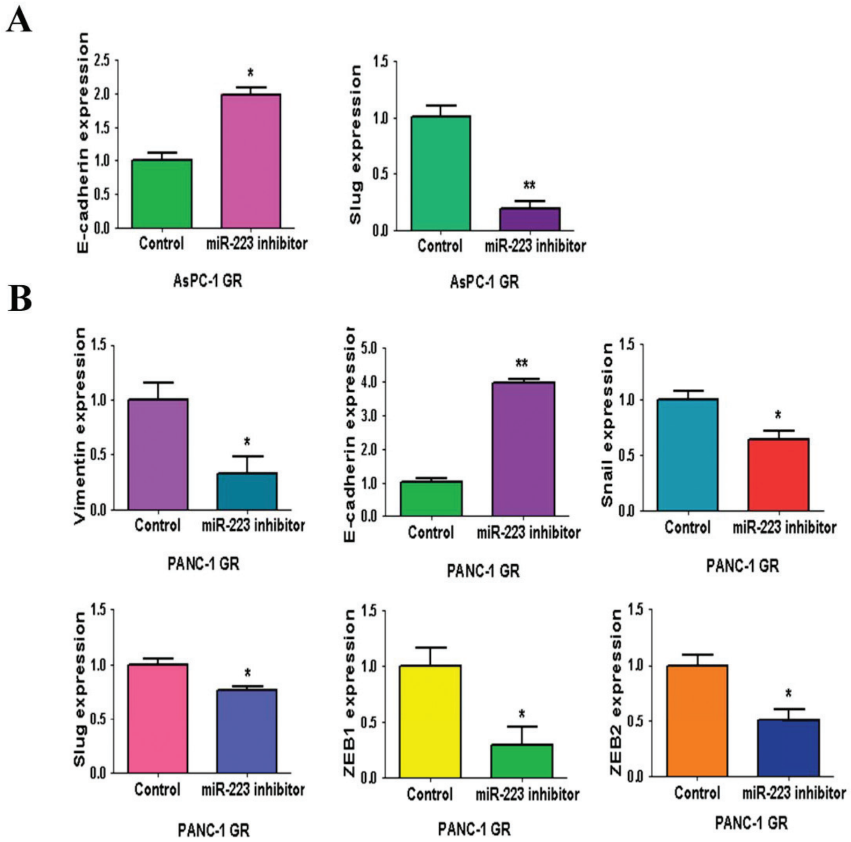

Supplementary Figure 1: Western blotting analysis was performed to detect the expression of EMT markers in AsPC-1 GR and PANC-1 GR cells after miR-223 inhibitor treatment in Figure 4D. Quantitative results are illustrated for cells treated with miR-223 inhibitor in AsPC-1 GR **(A)** and PANC-1 GR **(B)**. *$P < 0.05$ vs control; **$P < 0.01$ vs control.
